# From Association to Action: Personalizing Lifestyle Interventions in Multiple Sclerosis—A Critical Narrative Review

**DOI:** 10.3390/jpm16100523

**Published:** 2026-10-09

**Authors:** Ana Belén Caminero

**Affiliations:** Neurology Department, Hospital Nuestra Señora de Sonsoles, 05004 Ávila, Spain; caminero.nrl@gmail.com

**Keywords:** multiple sclerosis, lifestyle, personalized medicine, clinical decision-making, target engagement, behavior change, modifiable risk factors

## Abstract

Lifestyle factors are increasingly incorporated into multiple sclerosis (MS) care, but association, modifiability and therapeutic justification are not equivalent. This critical narrative review examines when a lifestyle exposure should become a clinical target after MS diagnosis. Evidence across physical activity and exercise, smoking, obesity and cardiometabolic health, diet, sleep, psychological stress, vitamin D and sun exposure, cognitive reserve and engagement, and alcohol is interpreted through the sequence modifiable exposure → post-diagnostic prognostic relevance → deliberate intervention → verified target modification (target engagement) → demonstrated clinical benefit, while distinguishing general-health benefit, MS-relevant clinical benefit and evidence for MS disease-course modification. The strength and completeness of this evidence vary substantially across domains. Smoking cessation and physical activity provide particularly strong grounds for clinical action, whereas several other exposures show larger gaps between prognostic association and intervention evidence. Multidomain intervention is not inherently personalized; personalization requires selection and prioritization of targets according to the intended outcome, strength of evidence, individual relevance and feasibility, followed by verification that the target changed and that the expected benefit occurred. Lifestyle intervention should complement, not substitute for, disease-modifying therapy, and no MS-directed intervention may be the appropriate personalized decision when a clinically relevant or sufficiently supported target is absent. The objective is therefore to support proportionate lifestyle decisions in which the intervention, intended outcome and therapeutic claim are matched to the available evidence and the individual clinical context.

## 1. Introduction: Lifestyle Has Entered MS Care, but Clinical Translation Remains Unresolved

The therapeutic landscape of multiple sclerosis (MS) has changed substantially over recent decades. Modern disease-modifying therapies (DMTs) can markedly suppress relapses and focal inflammatory disease activity, yet disability worsening remains a major therapeutic challenge, reflecting variable contributions from residual inflammation, compartmentalized central nervous system pathology, comorbid conditions and biological aging across individuals and disease stages [1]. Long-term MS management is consequently extending beyond control of acute inflammatory activity toward broader strategies that increasingly incorporate lifestyle and comorbidity management [1].

Potentially modifiable lifestyle factors have therefore moved into the clinical discourse surrounding MS. Smoking, physical inactivity, obesity and cardiometabolic health, diet, sleep, stress, vitamin D and sun exposure, among other factors, have been investigated in relation to MS susceptibility and subsequent outcomes, although the quantity and strength of evidence differ markedly across exposures [2]. Lifestyle recommendations have also entered brain-health-focused MS care. Recent work has established consensus recommendations and behavior-change strategies [3], while systematic assessments of MS guidelines have highlighted limitations in applicability and implementation resources [4]. More recently, Marck et al. evaluated the behavioral actionability of physical-activity recommendations using the AACTT framework—Actor, Action, Context, Target and Time—showing that even evidence-based recommendations may be insufficiently specified for implementation [5].

These advances address an important part of evidence translation, but leave a different clinical question unresolved: before asking how a lifestyle recommendation should be implemented, when does the evidence justify making that recommendation as a therapeutic intervention after MS diagnosis? Evidence that an exposure is associated with MS susceptibility does not establish its prognostic relevance after diagnosis; post-diagnostic association does not establish that deliberately modifying the exposure will improve outcomes; successful modification of the intended target does not ensure clinical benefit; and symptomatic, functional or general-health benefit does not necessarily imply modification of MS disease activity or progression. An ECTRIMS expert workshop identified substantially more evidence for lifestyle and environmental associations than for post-onset modification [2], while a systematic review of randomized interventions found no significant effect on MS progression for the interventions evaluated and emphasized limitations in study quality, size and duration [6].

This distinction separates the clinical actionability considered in this review from behavioral actionability or implementability. A recommendation may be precisely formulated in terms of who should do what, when and in what context, yet remain therapeutically unjustified for a particular MS outcome. Conversely, an intervention may be clinically warranted despite unavoidable limitations in randomized evidence, as with smoking cessation. The prior question is therefore whether a modifiable exposure should become a clinical target for a particular patient, for a defined outcome, and with what level of therapeutic claim [2,6].

Population-level recommendations cannot answer that question alone. Clinical value depends on whether a relevant target is present, what outcome is being pursued, the strength and nature of the intervention evidence, and the individual clinical context. Disease stage, disability, symptoms, comorbidities and competing priorities may alter the expected value of intervention; feasibility, preferences and capacity for sustained change subsequently determine how an appropriate recommendation can be implemented. Even well-established physical-activity recommendations require adaptation to ability, preferences, safety, comorbidities and symptom fluctuations rather than uniform prescription [7].

This review examines the pathway from lifestyle evidence to post-diagnostic therapeutic decision-making. Major lifestyle domains are evaluated through the sequence modifiable exposure → post-diagnostic prognostic relevance → deliberate intervention → verified target modification (target engagement) → demonstrated clinical benefit, while distinguishing general-health benefit, MS-relevant clinical benefit and evidence for MS disease-course modification. The review then examines multidomain interventions, individualized prioritization, implementation and monitoring, and translates the resulting evidence into representative clinical decisions. Unlike reviews that primarily catalogue lifestyle associations, summarize domain-specific interventions or address how recommendations should be implemented, this review makes the post-diagnostic therapeutic claim itself the unit of analysis: whether a particular exposure should be deliberately modified in a particular person, for a predefined outcome, and how success should be verified.

## 2. Methods: Literature Search and Evidence Synthesis

This critical narrative review was informed by structured, iterative searches of PubMed/MEDLINE, targeted bibliographic searches and reference-list examination of key reviews and pivotal studies. Searches covered physical activity and exercise, smoking, obesity and weight management, diet, sleep, psychological stress, vitamin D and sun exposure, cognitive reserve and engagement, alcohol and multidomain interventions. Domain terms were combined with concepts for post-diagnostic prognosis, disability, relapse, MRI activity, symptoms, cognition, quality of life, deliberate intervention, randomized trials, longitudinal exposure change, target engagement, adherence, implementation, feasibility, maintenance, sustainability and personalization. Searches were updated through 1 September 2026, with no lower publication-date limit. The search architecture and evidence-selection rules are reported in Supplementary Methods S1.

Eligible evidence was selected for its contribution to a post-diagnostic evidence-to-action question. Priority was given to post-diagnostic longitudinal studies, analyses of exposure change, randomized or deliberate intervention studies, systematic reviews and meta-analyses, and implementation or maintenance studies. Susceptibility studies were retained only when needed to distinguish etiological plausibility from post-diagnostic actionability. Small mechanistic or biomarker studies were used to assess biological plausibility or target modification, not as substitutes for clinical outcomes. Selection favored directness to the clinical question, methodological robustness, consistency with the wider evidence base and the potential to materially alter interpretation. No formal meta-analysis was undertaken. Because this was a critical narrative rather than a systematic review, screening was not conducted through a prespecified PRISMA process and no formal risk-of-bias instrument or GRADE assessment was applied.

Evidence within each lifestyle domain was interpreted along five explicitly defined dimensions: (1) directness to the post-diagnostic question; (2) study design and the causal inference it permits; (3) consistency across studies; (4) demonstration and maintenance of modification of the intended target; and (5) the outcome actually measured. Evidence was then mapped onto the sequence modifiable exposure → post-diagnostic prognostic relevance → deliberate intervention → verified target modification (target engagement) → demonstrated clinical benefit. The resulting therapeutic claim was classified as general-health benefit, MS-relevant clinical benefit or evidence relevant to MS disease-course modification. The qualitative descriptors used in Table 1 summarize these dimensions and are not presented as a formal GRADE certainty rating.

Where available evidence did not directly resolve a clinical decision, pragmatic recommendations are explicitly presented as the author’s clinical synthesis rather than as evidence-based directives. The evidence-to-action, prioritization and monitoring approach proposed in this review is therefore a conceptual clinical framework derived from the evidence synthesis and has not been prospectively validated as a clinical decision algorithm.

## 3. When Does a Modifiable Lifestyle Factor Become a Clinical Target?

Modifiability alone does not create a treatment indication. For a lifestyle exposure to become a clinical target after MS diagnosis, several distinct questions must be separated: Is the exposure associated with subsequent outcomes in established MS? Can it be deliberately modified? Was the intended target actually changed? Does that change produce a clinically meaningful benefit? And what therapeutic claim does the observed benefit support? Evidence is therefore interpreted through the sequence: modifiable exposure → post-diagnostic prognostic relevance → deliberate intervention → verified target modification (target engagement) → demonstrated clinical benefit. Clinical actionability arises from this evidence in relation to a particular patient and intended outcome; it is not an intrinsic property of the lifestyle factor itself.

### 3.1. Modifiability, Prognostic Relevance, Target Engagement and Therapeutic Actionability

A lifestyle factor may be modifiable because the exposure itself can be changed and prognostically relevant because it is associated with subsequent outcomes, without being therapeutically actionable for that outcome. The ECTRIMS focused workshop on environmental modifiable risk factors identified substantially more evidence relating lifestyle and environmental exposures to MS susceptibility than to post-onset disease activity or progression [2]. Similarly, a systematic review of randomized trials targeting potentially modifiable factors found no significant effect on MS progression for the interventions evaluated, while emphasizing limitations in study quality, size and duration [6].

Post-diagnostic longitudinal evidence narrows the causal gap but does not close it. In a large incident MS cohort followed for up to 15 years, higher physical activity at diagnosis and increases in activity thereafter were associated with more favorable subsequent disability outcomes [8]. This strengthens the prognostic relevance of physical activity after diagnosis, but does not establish that deliberately increasing activity will itself alter disability progression. Conversely, where randomized evidence is unethical or impractical, lack of an RCT does not make an intervention clinically unactionable; smoking cessation is the clearest example.

Target engagement adds a further necessary distinction. In this review, uptake means that an intervention is initiated or accepted; participation records exposure such as attendance, website use or program completion; adherence describes the extent to which the participant follows the agreed intervention; and fidelity describes whether the intervention is delivered as intended. None of these events alone establishes target engagement. Target engagement requires direct evidence that the behavioral, environmental, biochemical or physiological exposure specified by the therapeutic hypothesis actually changed and, when relevant, that the change was sustained. It is therefore closer to verification of the intervention’s intended proximal target than to engagement with the delivery process [9,10,11,12,13].

Clinical actionability in this review refers to whether the available evidence and individual clinical context justify deliberately modifying an exposure for a defined outcome. This differs from behavioral actionability as assessed by Marck et al. using AACTT, which concerns whether a recommendation adequately specifies the Actor, Action, Context, Target and Time required for implementation [5]. These concepts are complementary but not interchangeable: clinical justification precedes delivery; uptake, adherence and fidelity describe implementation; target engagement verifies that the intended exposure changed; and clinical benefit must then be demonstrated separately.

### 3.2. The Source of Evidence Determines the Therapeutic Claim

Susceptibility, prognosis and intervention studies provide complementary but therapeutically non-equivalent evidence. Etiological research implicating smoking, adolescent obesity, ultraviolet exposure or vitamin D-related pathways provides biological context but does not establish that modifying the same exposure after MS onset will alter disease trajectory [2]. The relevant causal window may precede diagnosis by years, and an exposure contributing to susceptibility cannot be assumed to remain reversible later in the disease course.

Post-diagnostic observational studies address a more clinically relevant but still non-interventional question. Obesity, for example, has been associated with disability accumulation and other adverse outcomes in established MS [14]. Such findings establish prognostic relevance more directly than susceptibility studies but do not demonstrate that intentional weight reduction improves those outcomes. Observational evidence also remains vulnerable to residual confounding and reverse causation; for physical activity, increasing disability may itself reduce activity and thereby exaggerate the apparent relationship between inactivity and subsequent disability [8].

Interventional evidence strengthens causal attribution only when both the intended exposure and the relevant outcome are adequately assessed. Randomization can establish the effect of offering an intervention, but if target engagement is absent or uncertain it cannot establish the effect of successfully modifying the target. Conversely, demonstration of behavioral, biochemical or physiological target engagement without a corresponding clinical outcome establishes that the exposure can be changed, not that changing it benefits MS [9,15].

Study design therefore determines how confidently an observed effect can be attributed to intervention, target-engagement measures determine whether the intended exposure was actually modified, and the selected outcome determines the therapeutic claim that can reasonably be made.

### 3.3. Clinical Benefit and MS Disease-Course Modification Are Not Equivalent

Lifestyle interventions may produce worthwhile benefits without modifying the biological course of MS. This review distinguishes three levels of benefit: (1) general-health benefit, including cardiovascular, metabolic, musculoskeletal and psychological health; (2) MS-relevant clinical benefit, including fatigue, cognition, mood, sleep, mobility, function, participation and quality of life; and (3) MS disease-course modification. The third category is an operational definition proposed for this review rather than a new consensus definition. It is reserved for sustained effects on outcomes that directly reflect MS disease evolution—such as relapses, inflammatory MRI activity or disability accumulation/progression—at a magnitude and duration appropriate to the claim. MRI effects may support modification of inflammatory disease activity without proving sustained clinical benefit, and biomarkers of neuroaxonal injury provide mechanistic or prognostic support but do not constitute disease-course modification by themselves.

Exercise illustrates why these levels should remain separate. MS-specific recommendations support exercise and lifestyle physical activity across the disability spectrum based on safety and benefits for fitness, function and selected MS-related outcomes, with individualized adaptation to ability, comorbidities and symptoms [7]. These benefits can justify intervention without requiring exercise to be classified as a disease-modifying therapy. The same principle applies across lifestyle domains: absence of demonstrated disease-course modification does not invalidate a reproducible symptomatic, functional or general-health benefit, but such benefit should not be relabelled as modification of MS biology or progression.

The converse is equally important. Prognostic association, biological plausibility or favorable biomarker change should not be used to promise a clinical benefit that has not been demonstrated. Calibrating the claim to the outcome prevents both therapeutic nihilism and therapeutic overstatement.

### 3.4. Actionability Is Patient-, Target- and Outcome-Specific

Whether an exposure should become a clinical target depends not only on the overall evidence but on its relevance to a particular patient and intended outcome. A well-supported intervention may have little value when the target is absent, already optimized or irrelevant to the patient’s priorities; a modest intervention effect may be worthwhile when the target is important, the expected benefit meaningful and the burden low. Conversely, modifiability and prognostic association alone may justify no MS-directed intervention [7,16].

The evidential threshold for recommending an intervention should also be proportionate to its burden, cost, restrictiveness and potential for harm, as well as to the magnitude of the therapeutic claim being made. For low-burden, low-risk behaviors with established general-health benefits, limited MS-specific evidence may constrain the claim that can be made rather than preclude the recommendation itself. Conversely, interventions that are restrictive, costly, intensive or potentially harmful require stronger evidence before being recommended for an MS-specific purpose. The evidential threshold for recommending an intervention is therefore not necessarily the same as that required for claiming an MS-specific therapeutic benefit.

The possibility of recommending no intervention is essential. Alcohol provides a useful example: observational associations between low or moderate consumption and more favorable MS outcomes do not establish benefit from initiating or increasing alcohol consumption, and therefore do not create a therapeutic indication. Clinical actionability must permit a conclusion of no intervention rather than converting every potentially favorable association into a prescription [17,18].

For the purposes of this review, the decision to intervene therefore rests primarily on four questions: is a relevant modifiable target present; is deliberate modification sufficiently supported for the intended outcome; is the expected benefit meaningful for this patient; and can target modification and the relevant outcome be assessed. Feasibility, patient preference, barriers, mode of delivery and maintenance then determine how a justified intervention should be implemented and are addressed in Section 6.

Personalization consequently means prioritization, not accumulation of recommendations. The objective is not to move every person with MS toward an idealized lifestyle profile, but to identify which target, if any, offers sufficient expected value to warrant intervention and what can reasonably be promised if that target is successfully modified. This framework underpins the domain-specific assessment in Section 4 and the subsequent translation into individualized clinical decisions [7,12,16]. Figure 1 summarizes this framework. Personalization as used in this review should not be equated with precision medicine in its stricter predictive sense. Current evidence for lifestyle interventions in MS generally does not permit validated estimation of individual treatment effects or reliable identification of predictive effect modifiers. Personalization therefore refers here to individualized clinical decision-making: identifying whether a relevant modifiable target is present, matching the strength and type of evidence to the intended outcome, and integrating this with clinical context, patient preferences, feasibility, burden and potential harm. Demonstration of heterogeneity of treatment effects and validated predictors of individual response would represent a further step toward precision lifestyle medicine and remains an important research need.

## 4. What Does the Evidence Allow Us to Act on?

Applying this framework reveals substantial heterogeneity across lifestyle domains. Some exposures have strong post-diagnostic prognostic associations but limited intervention evidence, whereas others have reproducible symptomatic, functional or general-health benefits despite uncertain effects on MS disease course.

A recent trajectory analysis illustrates this heterogeneity within a common post-diagnostic framework. Among 3163 newly diagnosed patients, smoking, obesity and low sun exposure were associated with a greater likelihood of unfavorable long-term EDSS trajectories, whereas higher physical activity was associated with a lower likelihood; baseline vitamin D levels were not significantly associated with trajectory membership [19]. As observational evidence, these findings strengthen post-diagnostic prognostic relevance but do not establish the effects of deliberate modification.

The “Clinical implications” subsections that follow represent the author’s evidence-informed interpretation and clinical synthesis of the available evidence within the framework proposed in this review. They should not be interpreted as formally graded treatment recommendations or consensus statements.

### 4.1. Physical Activity and Exercise

Physical activity and exercise are among the most extensively studied and clinically actionable lifestyle domains in MS. Meta-analyses and systematic reviews support benefits for health-related quality of life and walking mobility [20,21], balance [22], fitness, mobility, fatigue and quality of life [23], fatigue [24], and depressive symptoms [25]. Exercise training is generally safe in MS [26], although effect sizes and certainty vary across outcomes and cognitive effects are less consistent [27]. Physical inactivity remains common in MS [28], and MS-specific recommendations endorse exercise and lifestyle physical activity across the disability spectrum, individualized according to ability, safety, symptoms, comorbidities and preferences [7,29,30,31,32].

Post-diagnostic observational evidence also suggests prognostic relevance. Among 3284 people with incident MS followed for up to 15 years, higher physical activity at diagnosis and increases in activity thereafter were associated with more favorable subsequent disability outcomes [8]. These findings are clinically relevant because they concern activity after or around diagnosis, but residual confounding and reverse causation remain important: less aggressive disease may itself preserve the capacity to remain active.

Evidence for MS disease-course modification is substantially less secure. Exercise has plausible neuroplastic, neurotrophic, immunological and cardiovascular effects, and studies have reported changes in brain structure, functional connectivity, MRI measures and biological markers [33,34,35,36,37,38,39]. These findings support biological plausibility but do not establish sustained prevention of inflammatory disease activity or disability progression [40]. Similarly, completion of a supervised exercise program should not be assumed to increase habitual activity outside the intervention; where real-world physical activity is the intended target, target engagement should be demonstrated rather than inferred.

Heat sensitivity may transiently exacerbate pre-existing neurological symptoms during exercise and become an important barrier to participation. Exercise modality, intensity and format can be adapted, and cooling strategies may reduce exercise-induced heat sensitivity in susceptible individuals [41]. Such transient worsening should be distinguished clinically from new neurological disease activity. Evidence is also less secure at the highest levels of disability: a recent systematic review identified 29 studies but only six conducted exclusively in people with EDSS ≥6.5, limiting confidence in extrapolation from less disabled populations [42].

Clinical implication. Insufficient physical activity or a potentially responsive functional or symptomatic problem should prompt an individualized activity or exercise target. Exercise can be strongly recommended for fitness, walking and mobility, balance, fatigue, depressive symptoms, health-related quality of life and general health [20,21,22,23,24,25,26,31,43]. Recommendations should be adapted to safety, disability, symptoms and preferences [7,26,29,32,41,42]. These benefits are substantially better established than sustained prevention of inflammatory disease activity or long-term disability progression [30,33,34,35,36,37,38,39,40].

### 4.2. Smoking Cessation

Smoking is one of the clearest examples of a lifestyle exposure for which post-diagnostic prognostic evidence is sufficiently consistent to justify clinical action despite the absence of randomized cessation trials. Longitudinal studies and evidence syntheses associate continued smoking after MS onset with greater disability accumulation, faster progression and other adverse outcomes [44,45,46,47,48,49,50,51,52]. More recent MS-specific longitudinal evidence suggests more favorable subsequent disability outcomes after smoking cessation or among former compared with current smokers [50,51].

Randomized evidence assigning people with MS to continue smoking would be neither ethical nor clinically necessary. The therapeutic rationale rests on consistent post-diagnostic prognostic evidence [44,45,46,47,48,49,50,51,52], the established general-health benefits of cessation and guideline-supported cessation treatment [53]. MS-specific barriers and delivery limitations affect implementation [54,55,56,57], but do not negate the indication to stop smoking. Nevertheless, cessation should not be represented as randomized proof of modification of the biological course of MS.

Clinical implication. Current smoking should be treated as a priority modifiable target. The recommendation should be active cessation, supported where needed by behavioral and pharmacological strategies and attention to MS-specific barriers [53,54,55,56,57]. Clinicians can explain that stopping smoking has major general-health benefits and is associated with more favorable subsequent MS outcomes [44,45,46,47,48,49,50,51,52], while avoiding a guaranteed claim of prevention of disability progression.

### 4.3. Obesity and Cardiometabolic Health

Obesity is associated with less favorable disease activity, disability, cognition and quality-of-life outcomes after MS diagnosis [14,58]. In a pooled analysis of 16,794 participants from 17 phase III MS trials, increasing comorbidity burden was associated with greater disease activity, including an adjusted hazard ratio of 1.21 for evidence of disease activity among participants with at least two cardiometabolic conditions [59]. These findings strengthen the clinical relevance of cardiometabolic health but do not demonstrate that treating obesity or comorbidity burden itself reduces MS disease activity.

Smoking and obesity may also coexist in ways that increase prognostic burden. Recent longitudinal evidence identified additive interaction between smoking and obesity for MS-related outcomes [60]. This supports assessment of clustered risk rather than isolated exposures, but does not demonstrate therapeutic synergy from simultaneously modifying both targets.

A randomized behavioral weight-loss intervention demonstrated that diet and exercise targets and body weight can be modified in adults with MS and obesity [61]; a secondary analysis examined neurofilament light chain change after that intervention [62]. These findings address intervention feasibility and target modification, not the prognostic effect of baseline obesity. Evidence that intentional weight reduction modifies MS disease activity or long-term disability remains limited. Bariatric-surgery data require particular caution because surgery changes nutritional, metabolic and physiological exposures beyond body weight alone, and observational findings cannot be interpreted as the causal effect of weight reduction itself [63].

Clinical implication. Obesity and cardiometabolic risk should be addressed when present, with goals defined according to the individual clinical problem—weight reduction, metabolic risk, mobility, fatigability or general health—rather than presented as established MS disease-modifying treatment. Post-diagnostic associations support clinical relevance [14,58,59,60], whereas deliberate weight-loss evidence primarily supports feasibility, target modification and selected health or functional outcomes [61,62,63]. Coexisting smoking, hypertension, dyslipidemia, diabetes or other cardiometabolic targets may increase the priority of intervention, but the expected benefit should remain matched to the evidence.

### 4.4. Diet

Diet is among the lifestyle domains most vulnerable to overinterpretation because observational dietary patterns, deliberate dietary interventions and changes in metabolic or inflammatory biomarkers are often discussed as if they provided equivalent evidence. Observational studies have associated healthier dietary patterns with selected MS outcomes, while intervention studies have evaluated low-fat, Mediterranean-style, ketogenic, fasting or calorie-restriction strategies, among others [64,65,66,67,68,69,70,71]. Heterogeneity in dietary definitions, comparators, adherence, study duration and outcomes limits direct comparison.

Randomized trials [67,70,71] and evidence syntheses of randomized dietary interventions [68,69] indicate that dietary behavior and related metabolic targets can be modified and may improve selected symptoms, quality-of-life measures or cardiometabolic outcomes, but consistent evidence for MS disease-course modification is lacking. In a recent randomized trial of 105 people with RRMS, fasting and ketogenic strategies did not reduce the primary outcome of new T2 lesions compared with a standard healthy diet, although exploratory differences were reported in neurofilament light chain, cognition, depressive symptoms and cardiometabolic measures [71]. Such findings may generate hypotheses but should not be interpreted as demonstrated MS disease modification.

Clinical implication. A nutritionally adequate, high-quality diet can be recommended for general and cardiometabolic health, and specific dietary interventions may be considered when they address an individual target and are feasible and sustainable. Current evidence does not justify prescribing a specific dietary pattern as an established disease-modifying treatment for MS [64,65,66,67,68,69,70,71].

### 4.5. Sleep

Sleep disturbance is common in MS and is associated with fatigue, mood, cognition and health-related quality of life, but “poor sleep” should not be treated as a single modifiable exposure. Insomnia, sleep-disordered breathing, restless legs syndrome, circadian disturbance, nocturia, pain, spasticity, medication effects and behavioral sleep disruption may require different interventions and may also reflect rather than cause greater disease or symptom burden [72,73].

Intervention evidence is strongest for treating identifiable sleep problems rather than for sleep modification as a strategy to alter MS disease course. Randomized studies of cognitive behavioral therapy for insomnia have demonstrated improvements in insomnia severity and related patient-reported outcomes [74,75]. Recent systematic and meta-analytic evidence likewise supports improvement in subjective sleep outcomes with non-pharmacological interventions, although the interventions and sleep problems studied were heterogeneous [76,77]. Together, these findings support mechanism-specific assessment and symptomatic treatment rather than generic sleep modification as an MS disease-modifying strategy [73,77].

Clinical implication. Sleep should become a treatment target when a clinically relevant and potentially modifiable sleep problem is identified. The appropriate intervention depends on its mechanism, and benefit should be assessed through the corresponding sleep, fatigue, daytime function or quality-of-life outcome. Current evidence supports symptomatic and functional benefit rather than modification of MS inflammatory activity or disability progression [72,73,74,75,76,77].

### 4.6. Psychological Stress

Psychological stress is biologically plausible as a modifier of MS-related outcomes, but the clinical evidence is heterogeneous and vulnerable to differences in stress definition, measurement, timing and confounding. Systematic and meta-analytic evidence suggests a small-to-modest association between psychological stress and subsequent MS disease activity, but does not establish a large or deterministic causal effect [78,79].

Stress-management interventions provide a clearer basis for symptomatic and psychological action. Systematic-review evidence [80], an individual randomized trial [81], and subsequent meta-analytic or systematic syntheses [82,83] report benefits in stress, mood, coping or quality of life. A randomized stress-management trial also demonstrated fewer new MRI lesions during active treatment, but the radiological effect was not sustained after treatment ended and no corresponding effect on clinical relapses or disability was established [84].

Clinical implication. Stress management is appropriate when stress itself, coping difficulties or associated psychological symptoms constitute clinically relevant targets. The expected benefit should be framed primarily in terms of psychological well-being, coping and quality of life. Current evidence does not justify promising prevention of relapses or disability progression through stress reduction [78,79,80,81,82,83,84].

### 4.7. Vitamin D and Sun Exposure

Vitamin D and sun exposure illustrate particularly well the distinction between susceptibility evidence, post-diagnostic prognosis and intervention. Susceptibility and mechanistic studies link lower vitamin D status and lower sun or ultraviolet radiation exposure with MS risk [85,86,87,88,89,90], while post-diagnostic observational studies associate these exposures with inflammatory activity or subsequent outcomes [91,92,93,94,95,96]. Circulating vitamin D and UVR exposure should not be treated as interchangeable because their associations may not be mediated through the same biological pathway.

Vitamin D supplementation trials have produced less consistent results than observational studies [15,97,98,99,100,101], a conclusion also reflected in systematic and meta-analytic evidence [102]. Importantly, these data demonstrate that biochemical target engagement does not guarantee clinical benefit. In VIDAMS, high-dose vitamin D3 produced the intended increase in circulating vitamin D levels but did not significantly reduce relapse risk or provide consistent clinical or MRI benefit compared with low-dose supplementation [15].

More recent evidence prevents an entirely negative interpretation. In D-Lay MS, high-dose cholecalciferol administered after a recent clinically isolated syndrome reduced the composite primary disease-activity endpoint over 24 months (60.3% vs. 74.1%; HR 0.66, 95% CI 0.50–0.87), accompanied by significant effects on MRI outcomes [103]. However, relapse and other clinical secondary outcomes were not significantly improved. Recent quantitative syntheses have likewise reached partly discordant conclusions regarding relapse, MRI and disability outcomes [102,104,105,106]. Differences in disease stage, baseline vitamin D status, dose, comparator exposure and trial duration may contribute to this heterogeneity.

Trial design and background therapy should also be considered when interpreting these apparently discordant results. Several earlier trials evaluated vitamin D supplementation as an adjunct to ongoing immunomodulatory therapy, which may reduce inflammatory event rates and make an additional treatment effect more difficult to detect. By contrast, D-Lay evaluated high-dose cholecalciferol in untreated patients after a recent clinically isolated syndrome, thereby addressing a different therapeutic setting. This distinction may partly contribute to differences across trials, although it does not establish background DMT as the explanation for previous negative findings.

Clinical implication. Vitamin D deficiency or insufficiency may reasonably be corrected according to established health indications, and supplementation is a modifiable biological target that can be monitored directly. Post-diagnostic associations provide prognostic context [91,92,93,94,95,96], but supplementation trials remain heterogeneous [15,97,98,99,100,101,102,103] and recent quantitative syntheses are partly discordant [102,104,105,106]. Current evidence does not establish a reproducible vitamin D regimen, serum target or UVR prescription that can be recommended as an MS disease-modifying treatment. Positive MRI-dominated findings warrant continued investigation but should not be translated into a guaranteed clinical benefit.

### 4.8. Cognitive Reserve and Engagement

Cognitive reserve is associated with resilience to cognitive impairment in MS. Observational longitudinal studies link premorbid intellectual enrichment and reserve-related characteristics with differences in cognitive outcomes and symptom experience [107,108,109,110]. These findings support cognitive reserve as a prognostic or explanatory construct, but reserve itself is not a directly manipulable exposure and observational associations do not demonstrate that increasing cognitive activity after diagnosis will build reserve or prevent cognitive decline.

Deliberate cognitive engagement is therefore a related but distinct therapeutic question. Small intervention studies indicate that cognitively stimulating activity can be increased, demonstrating behavioral target engagement, but evidence for sustained objective cognitive benefit remains limited. In a 12-week randomized trial of 60 people with MS, increasing cognitively stimulating leisure activities did not significantly improve the co-primary objective cognitive outcome measured by the Symbol Digit Modalities Test. A between-group difference was observed in subjective cognition, but this was driven primarily by worsening in the control group rather than clear objective cognitive improvement in the intervention group [111].

Cognitive rehabilitation should also be distinguished from lifestyle cognitive engagement. Structured cognitive rehabilitation may be clinically appropriate for established cognitive impairment, but evidence supporting such treatment does not demonstrate that cognitively stimulating lifestyle activity prevents future cognitive decline by increasing cognitive reserve [112].

Clinical implication. Maintaining meaningful cognitive, social and intellectually stimulating activity is reasonable for participation, well-being and general brain health. Intervention studies show that cognitive engagement can be delivered and increased, but sustained objective preventive efficacy remains unproven [111,113]. Cognitive rehabilitation addresses established impairment and a different therapeutic target [112]. Current evidence therefore does not establish cognitively stimulating lifestyle activity as a proven means of building cognitive reserve, preventing MS-related cognitive decline or modifying MS disease course.

### 4.9. Alcohol

Alcohol provides a useful counterexample to the assumption that every favorable observational association should generate a therapeutic recommendation. Prospective and longitudinal studies have reported associations between low or moderate alcohol consumption and selected more favorable MS outcomes [17,18]. These findings remain susceptible to residual confounding, differences between abstainers and drinkers, reverse causation and changes in consumption after illness.

Available MS evidence remains observational, with no established therapeutic dose or target for initiating or increasing alcohol consumption [17,18]. These associations therefore do not justify translation into an MS-directed prescription, particularly given alcohol’s potential harms.

Clinical implication. Alcohol consumption should not be initiated or increased as an MS intervention. Advice should follow general-health recommendations and individual clinical circumstances. In the evidence-to-action framework, alcohol illustrates an important personalized conclusion: a modifiable exposure may have prognostic associations without creating a therapeutic target [17,18].

## 5. Beyond Single Factors: Multidomain Does Not Mean Personalized

Lifestyle behaviors frequently cluster, and observational studies suggest that combined lifestyle profiles may be associated with clinically relevant outcomes in MS [114,115,116]. Such findings support considering lifestyle domains jointly, but they do not establish that simultaneously modifying several behaviors improves MS disease course.

Multidomain interventions have demonstrated feasibility and benefits across selected behavioral, symptomatic, cognitive or quality-of-life outcomes [117,118,119,120]. However, intervention delivery and participation do not demonstrate that all intended lifestyle targets were modified, nor do improvements after a complex intervention identify which component produced the benefit. Where target modification is measured, it should therefore be interpreted separately from clinical benefit.

Outcome-specific benefit is also illustrated by the 2026 pragmatic LAMONT trial. Among 470 people with MS selected for impaired quality of life, levidex added to treatment as usual improved MS-specific quality of life at six months compared with treatment as usual alone (baseline-adjusted mean difference in HAQUAMS −0.10, 95% CI −0.18 to −0.03; *p* = 0.008), and clinically relevant improvement occurred in 39.5% versus 27.8% of participants (number needed to treat 9). Benefits were also observed for depressive symptoms and social/work functioning, but not anxiety. These findings support matching a complex intervention to a demonstrably impaired baseline outcome rather than inferring universal or disease-modifying benefit [121].

More individualized multidomain approaches illustrate both the potential and the limitations of personalization. In POWER@MS1, the personalized digital levidex intervention did not significantly prolong time to first new T2 lesion and/or relapse compared with an active comparator providing lifestyle information (HR 0.91, 95% CI 0.66–1.27), and sustained between-group differences were not demonstrated for most targeted behaviors [9]. The trial therefore does not show that lifestyle intervention is ineffective; rather, it illustrates the difficulty of demonstrating added benefit from a more intensive personalized intervention when the comparator itself contains potentially active lifestyle information.

A multidomain intervention is not inherently personalized. Its value depends on whether each component addresses a present and sufficiently supported target, whether the intervention burden is acceptable and whether the intended targets and outcomes are measured. Several domains may appropriately be addressed together, but breadth should follow from clinical priorities rather than from an assumption that more components are necessarily better [9,114,115,116,117,118,119,120]. Table 1 summarizes the evidence-to-action assessment across the major lifestyle domains discussed above.

**Table 1:** Evidence-to-action assessment of major lifestyle domains after MS diagnosis.

Lifestyle Domain	Post-Diagnostic Relevance	Intervention and Target Engagement	Demonstrated Benefit	MS Disease-Course Modification	Author’s Evidence-Informed Clinical Translation	References
**Physical activity/exercise**	Higher activity and increases after diagnosis associated with more favorable disability outcomes; reverse causation remains possible.	Extensive RCT evidence; fitness/function modifiable. Habitual activity should be measured when it is the intended target.	Fitness, mobility, selected fatigue/mood/HRQoL; cognition less consistent.	Biological/MRI plausibility, but sustained prevention of inflammatory activity or disability progression not established; evidence less secure at very high disability.	Act when inactivity or a responsive clinical target is present; individualize modality, intensity, disability and heat management.	[7,8,20,21,22,23,24,25,26,27,29,30,31,32,33,34,35,36,37,38,39,40,41,42,43]
**Smoking**	Continued smoking associated with adverse outcomes; cessation/former smoking with more favorable subsequent trajectories.	Directly modifiable; randomized continued-smoking trials unethical; implementation barriers documented.	Major general-health benefit; longitudinal MS evidence supports cessation.	No randomized proof, but observational evidence is sufficient to act.	Priority target; active cessation and support.	[44,45,46,47,48,49,50,51,52,53,54,55,56,57]
**Obesity/cardiometabolic**	Obesity and cardiometabolic burden associated with less favorable outcomes; clustered risk with smoking.	Weight and metabolic targets are modifiable; intervention evidence less extensive.	Weight/metabolic and selected functional/QoL benefits.	Insufficient evidence that intentional weight loss modifies MS disease course.	Act when present with defined weight/metabolic/functional goals; avoid disease-modifying claims.	[14,58,59,60,61,62,63]
**Diet**	Healthier patterns associated with selected outcomes, with confounding.	Dietary targets can be modified; multiple heterogeneous RCTs.	Selected fatigue/QoL/metabolic outcomes.	No specific diet reproducibly disease modifying.	Recommend nutritional adequacy and diet quality; not a branded “MS diet”.	[64,65,66,67,68,69,70,71]
**Sleep**	Sleep disorders common and associated with fatigue, mood, cognition and QoL; relationships may be bidirectional.	Identifiable disorders are modifiable; CBT-I and other non-pharmacological interventions improve sleep outcomes.	Sleep and daytime symptom/function benefits.	No disease-course modification evidence.	Identify the disorder/mechanism and target the corresponding outcome.	[72,73,74,75,76,77]
**Stress**	Small-to-modest association with MS activity/outcomes; heterogeneous.	Stress-management and mindfulness interventions reduce psychological burden.	Stress, mood, coping and QoL; transient MRI signals in one RCT.	No sustained relapse/disability benefit established.	Act when stress burden is clinically relevant; avoid causal blame or disease-modifying promises.	[78,79,80,81,82,83,84]
**Vitamin D/sun**	Lower vitamin D/sun exposure associated with susceptibility and selected post-diagnostic outcomes; not interchangeable exposures.	Supplementation reliably changes 25(OH)D; VIDAMS demonstrates target engagement without consistent clinical benefit.	Selected MRI signals; D-Lay positive composite/MRI result without significant clinical secondary outcomes.	RCTs/meta-analyses remain discordant; no reproducible regimen or serum target.	Correct deficiency; do not prescribe unvalidated high-dose or UVR treatment as MS disease modification.	[15,85,86,87,88,89,90,91,92,93,94,95,96,97,98,99,100,101,102,103,104,105,106]
**Cognitive reserve/engagement**	Reserve-related characteristics observationally favorable.	Engagement can be modified, but reserve itself is not directly manipulable.	Limited subjective/short-term cognitive signals; no consistent sustained objective benefit.	No prevention of cognitive decline or disease-course modification established.	Meaningful engagement is reasonable; distinguish from cognitive rehabilitation.	[107,108,109,110,111,112,113]
**Alcohol**	Low/moderate consumption associated with selected favorable outcomes observationally.	No therapeutic intervention or validated target/dose.	No demonstrated therapeutic benefit from deliberate modification.	Not established.	No MS-directed prescription; follow general-health guidance.	[17,18]

Note. Evidence is grouped by evidentiary function using full-text labels rather than letter codes. Reviews and primary studies are separated where they serve different functions. Interpretation is qualitative and domain-specific, not a formal GRADE rating. “Not established” indicates insufficient or inconsistent evidence, not proof of no effect. Clinical translation reflects the author’s evidence-informed interpretation and synthesis of the available literature and should not be understood as formally graded treatment recommendations or consensus guidance.

## 6. Making a Worthwhile Intervention Achievable

### 6.1. Specify the Action and the Outcome

A justified recommendation still requires translation into a specific action. Generic advice such as “exercise more”, “eat better”, “sleep better” or “reduce stress” leaves the behavior insufficiently defined for implementation or subsequent evaluation. Recent analysis of physical-activity guidelines using the AACTT framework showed that recommendations frequently omit important specification of Actor, Action, Context, Target or Time [5]. Structured goal-setting may improve the specificity and evaluability of patient goals in MS [122], although this should not be interpreted as evidence that goal-setting itself improves clinical outcomes.

The practical target should therefore specify what is expected to change and, where appropriate, by how much, under what circumstances and over what period. The target should also correspond to the intended therapeutic outcome. A supervised exercise program, for example, may target fitness or strength directly; if the intended intervention is to increase habitual physical activity, attendance at supervised sessions alone does not establish that the relevant target changed [5,122].

### 6.2. Adapt Intervention to the Person and Context

Even a clinically justified and well-specified target may fail if the intervention is incompatible with the person’s disability, symptoms, comorbidities, resources, preferences or daily circumstances. Studies of lifestyle management in MS identify barriers and facilitators across individual, interpersonal, healthcare and broader contextual levels [12], while shared decision-making emphasizes the importance of incorporating patient preferences and priorities [16]. Behavioral response may also differ according to clinical characteristics: in a physical-activity intervention, response varied according to MS course, disability and body mass index [123].

Qualitative and process-evaluation studies similarly emphasize tailoring, flexible delivery and appropriate professional support [124,125]. Digital delivery may reduce some access barriers and has demonstrated feasibility in MS lifestyle interventions [10,126], but digital access, cognitive burden, fatigue and the need for human support may themselves influence participation.

Smoking cessation illustrates the distinction between giving correct advice and making change achievable. People with MS and clinicians report individual barriers, competing priorities, variable confidence and uncertainty regarding cessation support or referral pathways [54,55,56,57]. MS-specific intervention development therefore increasingly incorporates these barriers rather than assuming that advice alone will produce cessation [57].

Personalization may sometimes require doing less, but choosing better. An intervention that addresses the highest-value target with an acceptable burden may be more useful than a comprehensive program that exceeds the patient’s capacity for sustained change. This is an evidence-informed clinical synthesis supported by implementation, shared-decision and intervention-response literature [10,12,16,123,124,125,126].

### 6.3. Verify Target Engagement, Maintenance and Benefit

Implementation should distinguish the proximal steps defined in Section 3.1. Uptake, participation, adherence and fidelity describe whether an intervention begins and how it is received or delivered; they do not by themselves show that the intended lifestyle exposure changed. Enrollment, attendance, website use or completion should therefore be interpreted as implementation measures rather than as evidence of target engagement [10,11,13].

Target engagement should therefore be assessed with a measure appropriate to the intervention: smoking status for cessation, body weight or relevant metabolic measures for weight management, serum 25-hydroxyvitamin D for supplementation, validated sleep measures for insomnia treatment, or habitual activity when increasing daily physical activity is the intended target. Monitoring should remain proportionate to the clinical decision; in the framework proposed here, the simplest measure capable of establishing whether the relevant target changed is generally preferable [10,11,15].

Clinical benefit should then be evaluated against the outcome that justified intervention. Lack of benefit despite verified and sustained target modification raises a different question from lack of benefit because the target never changed. Conversely, perceived benefit without demonstrated target modification may still be clinically valuable but should not automatically be attributed to the intended mechanism. Maintenance is a further consideration because behavioral changes achieved during structured interventions may not persist once active support is withdrawn [13].

The purpose of monitoring is therefore not to medicalize every lifestyle behavior or generate additional measurement burden. It is to determine whether a chosen intervention is worth continuing, adapting, replacing or de-escalating. Figure 2 summarizes this troubleshooting pathway as an evidence-informed clinical aid rather than a validated causal algorithm.

## 7. From Evidence to the Consultation: What Should the Neurologist Actually Do?

Lifestyle assessment becomes clinically useful only when it changes a decision. The consultation should therefore move beyond asking whether a behavior is “healthy” and address three questions: Is there a relevant modifiable target in this patient? What outcome would justify changing it? What can reasonably be expected if the target is successfully modified? [2,6,7,16].

These questions organize lifestyle issues according to clinical priority rather than as a universal checklist. A target merits priority when it is present, clinically relevant, sufficiently supported for the intended outcome and feasible at an acceptable burden. Some factors instead act as enabling targets—for example, insomnia, heat sensitivity, pain or environmental barriers that prevent physical activity—whereas already optimized behaviors may require only maintenance. Within the evidence-to-action framework proposed here, the author’s clinical synthesis is that when no sufficiently supported or relevant target is present, no MS-directed intervention may itself be the appropriate personalized decision.

The strength of the therapeutic claim should remain proportional to the evidence. Smoking cessation has major established health benefits and is associated with more favorable subsequent MS outcomes [44,45,46,47,48,49,50,51,52], without randomized proof of MS disease-course modification. Exercise has reproducible benefits for fitness, mobility, balance, fatigue, mood and quality of life [20,21,22,23,24,25,26,31,43], whereas evidence for sustained prevention of MS progression remains insufficient [30,33,34,35,36,37,38,39,40]. Treating insomnia can improve sleep and related daytime outcomes [74,75,76,77] without proving reduced MS activity; observational alcohol associations do not justify initiating or increasing consumption [17,18]; and vitamin D trials [15,97,98,99,100,101,102,103] and syntheses [102,104,105,106] do not establish a reproducible disease-modifying regimen.

Lifestyle interventions should complement rather than substitute for established MS treatment. Current evidence does not justify presenting lifestyle modification as an alternative to indicated DMT or promising prevention of progression independent of established disease biology and treatment [1,2,6,7].

Table 2 translates this approach into representative clinical scenarios. These examples are intended as an evidence-informed clinical synthesis, not formally graded treatment recommendations. Monitoring is included only where it can help determine whether the intended target changed, whether the expected benefit occurred and whether intervention should be continued, adapted or reconsidered.

## 8. What Evidence Is Still Needed?

### 8.1. Distinguish Prognostic Targets from Intervention Effects

The central evidence gap is not whether lifestyle exposures correlate with MS outcomes, but whether deliberately modifying a specific exposure after diagnosis produces a clinically meaningful and sustainable benefit. Expert assessment and systematic review have both documented the imbalance between association evidence and intervention evidence [2,6]. Post-diagnostic longitudinal studies strengthen prognostic relevance—for physical activity and disability [8], and for smoking, obesity, sun exposure and activity as predictors of long-term disability trajectories [19]—but they cannot establish the effect of deliberate modification.

Future studies should distinguish between identifying a prognostic target and testing its modification. Intervention studies are required when the therapeutic question is whether changing an exposure changes an outcome [2,6]. Where randomization is unethical or impractical, as for continued smoking, longitudinal analyses of exposure change and appropriately designed causal-inference studies remain necessary [44,45,46,47,48,49,50,51,52].

### 8.2. Measure Target Engagement

Intervention studies should establish whether the intended behavioral, biological or physiological target actually changed. Existing trials illustrate why this distinction matters. In VIDAMS, vitamin D supplementation produced biochemical target modification without a corresponding significant reduction in relapse risk or consistent clinical benefit [15]. Conversely, participation in complex behavioral interventions does not ensure sustained modification of every intended lifestyle target; POWER@MS1 did not demonstrate sustained between-group differences in most targeted behaviors compared with an active lifestyle-information comparator [9].

Attendance, program completion and other implementation metrics should not substitute for measurement of target modification. Target-engagement measures must correspond to the therapeutic hypothesis and establish whether the intended behavioral or biological exposure changed and, where relevant, whether the change was maintained [9,10,11,13,15].

### 8.3. Match Outcomes and Follow-Up to the Therapeutic Claim

Studies should be designed around the claim they are intended to test. Improvement in symptoms, function, quality of life or cardiometabolic health may be clinically valuable without establishing MS disease-course modification. Conversely, biological or MRI effects may provide mechanistic or disease-activity evidence without demonstrating sustained clinical benefit. D-Lay MS illustrates this distinction: high-dose cholecalciferol reduced the composite disease-activity endpoint and several MRI outcomes, while relapse and other clinical secondary outcomes were not significantly improved [103]. Similarly, a randomized stress-management intervention reduced new MRI lesion activity during treatment, but the radiological effect was not sustained after treatment ended, and no corresponding clinical benefit was established [84]. Trials intended to support claims regarding disability accumulation or progression therefore require outcomes, sample sizes and follow-up appropriate to those claims. Future studies should explicitly define both the intended clinical outcome and the magnitude and duration of target modification required to test the therapeutic hypothesis [6,84,103].

### 8.4. Study Heterogeneity of Response and Underrepresented Populations

Average intervention effects may conceal clinically important heterogeneity. Behavioral response to a physical-activity intervention has differed according to MS course, disability status and body mass index [123], while recent systematic review evidence highlights how few exercise studies have been conducted exclusively in people with severe disability [42]. These examples illustrate both potential heterogeneity of response and important gaps in the populations represented in intervention research.

Future trials should examine whether benefit depends on baseline target status, disease stage, disability, symptoms, comorbidity, age or other clinically plausible modifiers. Participants whose behavior or biological exposure is already optimized may have little opportunity for target modification, reducing both the plausibility of benefit and the ability of a trial to detect it. Such analyses should be explicitly planned and adequately powered where possible rather than generated through exploratory subgroup proliferation [42,123].

### 8.5. Identify What Adds Value in Complex Interventions

Complex and multidomain interventions create an additional challenge because an overall intervention effect does not identify which components were necessary, additive or redundant. Trials such as POWER@MS1 and their accompanying process evaluations illustrate the value of assessing intervention use, behavioral change, participant experience and implementation alongside clinical outcomes [9,124]. Broader MS lifestyle and self-management literature likewise identifies barriers operating at patient, interpersonal, healthcare and contextual levels and demonstrates that feasibility and completion cannot be assumed from efficacy alone [10,12,126].

Future multidomain and personalized interventions should therefore be designed, where feasible, to identify the incremental contribution of individual components while measuring uptake, target engagement, maintenance, burden and clinically relevant outcomes. The resources required for implementation should also be considered. The aim is not simply to demonstrate that lifestyle change is possible, but to determine what is worth changing, for whom, for what outcome, and at what burden [9,10,12,124,126].

## 9. Conclusions

This review has limitations inherent to its critical narrative design. Although the literature search and evidence-selection approach were structured and explicitly defined, study selection and interpretation necessarily involved author judgement, and no prespecified systematic-review process, formal risk-of-bias assessment or GRADE evaluation was undertaken. Accordingly, the proposed evidence-to-action framework and the domain-specific clinical implications should be interpreted as an evidence-informed clinical synthesis rather than as formally graded recommendations or a validated decision algorithm.

Lifestyle management has a legitimate place in comprehensive MS care, but the evidence supporting action differs substantially across exposures and outcomes. Prognostic association, modifiability, biological plausibility, uptake and target engagement should not be treated as equivalent to therapeutic benefit. Conversely, absence of demonstrated MS disease-course modification should not obscure reproducible symptomatic, functional or general-health benefits.

Personalized lifestyle care requires selection rather than accumulation of recommendations: a relevant target, sufficient justification for a predefined outcome, feasible delivery, verified target modification and separate assessment of clinical benefit. When these conditions are not met, adaptation, replacement, de-escalation or no MS-directed intervention may be the appropriate decision. The aim is neither a universal lifestyle package nor an alternative disease-modifying therapy, but proportionate decisions about what is worth changing now, for this person, and how we will know whether it was worth it.

## Figures and Tables

**Figure 1:**
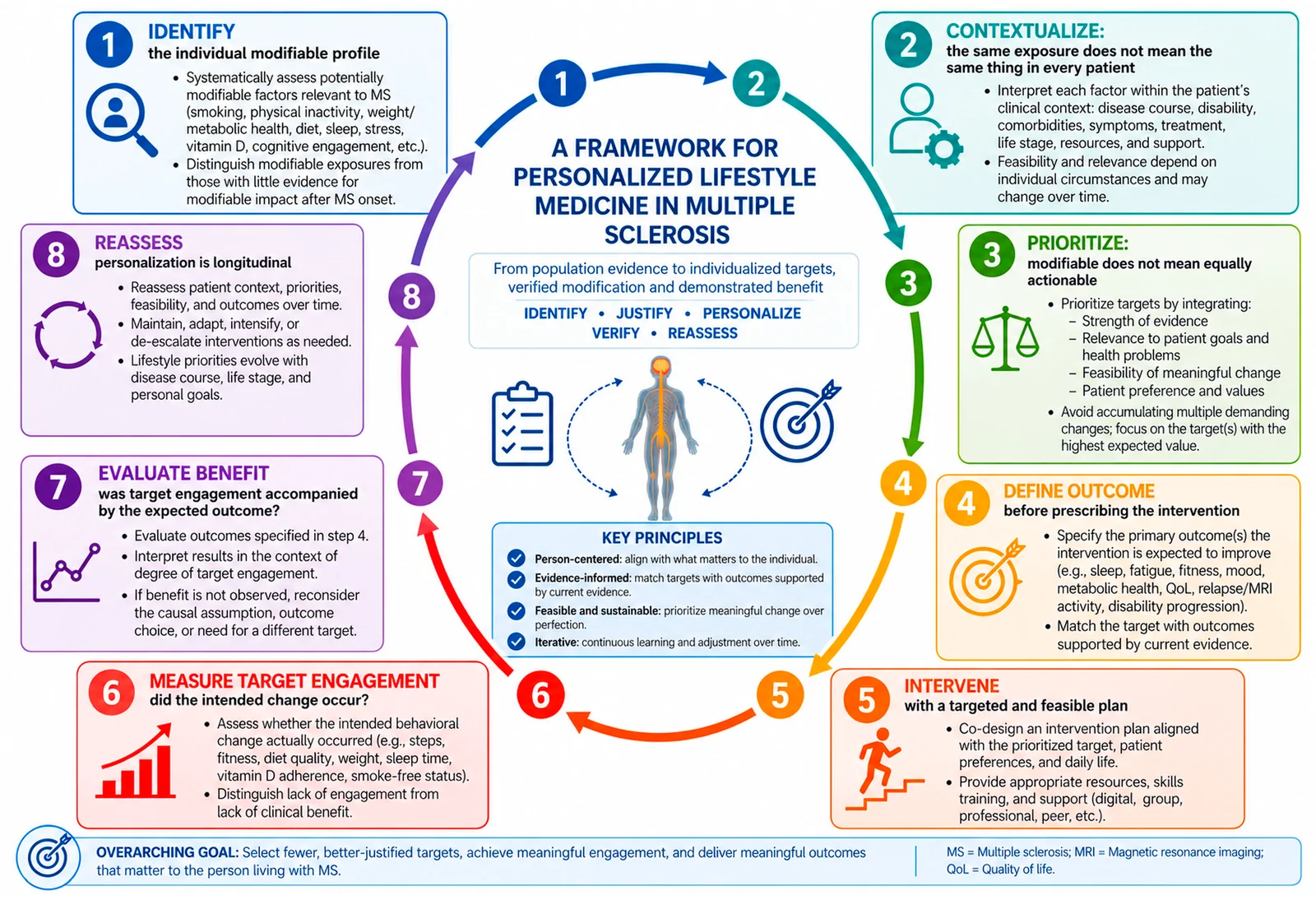
Proposed framework for personalized lifestyle medicine in multiple sclerosis. The framework integrates identification and contextualization of a relevant exposure, justification of intervention for a defined therapeutic outcome, individualized target prioritization and delivery, and separate verification of target modification and clinical benefit. Reassessment is longitudinal and may lead to maintenance, adaptation, intensification, target change, de-escalation or no intervention. Target engagement must be demonstrated but does not guarantee clinical benefit. This is an evidence-informed synthesis and has not been prospectively validated as a clinical decision algorithm.

**Figure 2:**
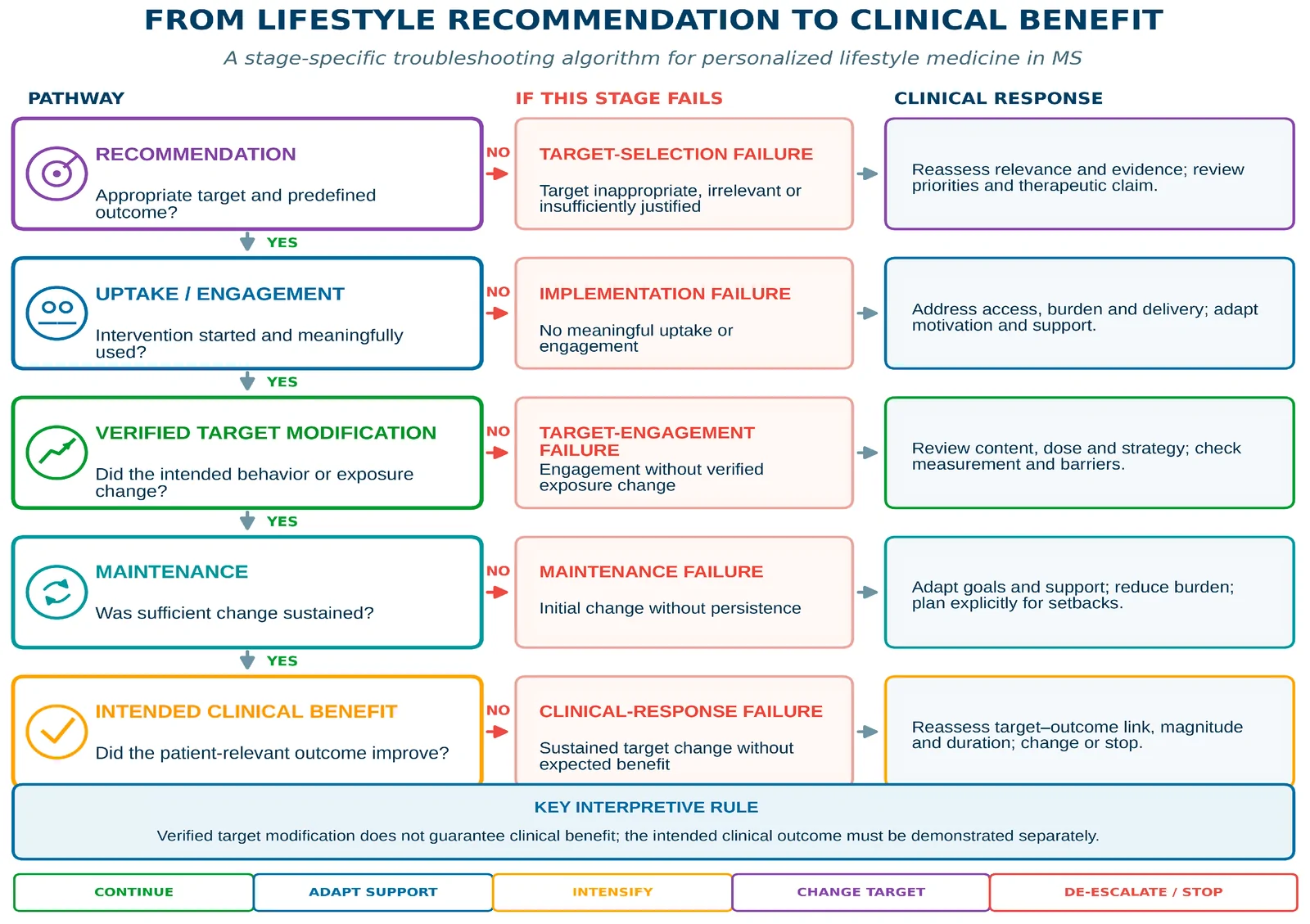
From lifestyle recommendation to clinical benefit: identifying where the pathway succeeds or fails. The pathway distinguishes target selection, intervention uptake and engagement, verified modification of the intended lifestyle target, maintenance and subsequent clinical benefit. Failure at each stage has different implications and should prompt a stage-specific response. Target engagement does not guarantee clinical benefit. This is a conceptual clinical aid, not a validated causal algorithm.

**Table 2:** Translating lifestyle evidence into individualized clinical action in multiple sclerosis.

Clinical Scenario	What to Prioritize	What to Avoid	Practical Target/Monitoring	How to Frame It	References
Current smoker	Active cessation with behavioral/pharmacological support as needed.	Generic advice without support; promises of reversal.	Sustained abstinence; reassess smoking status.	Cessation has major health benefits and is associated with more favorable subsequent MS trajectories; an MS-specific causal effect is not established.	[44,45,46,47,48,49,50,51,52,53,54,55,56,57]
Inactive without major limitation	Individualized increase in activity/exercise.	Presenting exercise as DMT.	Activity/fitness target plus patient-relevant outcome.	Strong evidence for fitness, mobility and selected symptoms; progression prevention unproven.	[7,8,20,21,22,23,24,25,26,29,30,31,32,33,34,35,36,37,38,39,40,43]
Inactivity driven by disability, fatigue or heat	Address enabling barriers; adapt modality/intensity and consider cooling.	Interpreting transient heat-related worsening as new disease activity.	Feasible activity target; symptom/barrier monitoring.	Adaptation can make activity achievable; severe-disability evidence is less secure.	[41,42]
Obesity + cardiometabolic risk	Weight/metabolic targets linked to health/function.	MS-specific weight-loss promises.	Weight/waist ± BP, glucose/lipids; function if relevant.	General/metabolic benefit; intentional weight loss not established as MS disease modification.	[14,58,59,60,61,62,63]
Obesity + smoking/multiple cardiometabolic risks	Prioritize highest-value risks; sequence if burden is high.	Assuming simultaneous change is necessarily superior.	One or few targets with explicit follow-up.	Clustered risk may increase priority; therapeutic synergy is unproven.	[59,60]
Asks for the “best MS diet”	Nutritionally adequate high-quality diet.	Restrictive branded diet as MS treatment.	Diet-quality/adherence and intended metabolic/symptom outcome.	No specific diet is established as disease modifying.	[64,65,66,67,68,69,70,71]
Insomnia/identifiable sleep disorder	Treat the specific disorder, e.g., CBT-I where appropriate.	Generic “sleep better” advice.	Validated sleep measure ± daytime function/fatigue.	Symptomatic/functional benefit; no proven disease-course modification.	[72,73,74,75,76,77]
High psychological stress	Evidence-based stress-management/psychological intervention.	Blaming stress for MS or promising relapse prevention.	Stress/coping/QoL outcome.	Psychological benefit is supported; disease-modifying claim is not.	[78,79,80,81,82,83,84]
Vitamin D deficiency	Correct deficiency according to established health indications.	Unvalidated MS serum target/high-dose prescription.	25(OH)D when clinically appropriate.	Correction is reasonable; MS-specific disease modification remains unproven.	[15,91,92,93,94,95,96,97,98,99,100,101,102,103,104,105,106]
Adequate vitamin D but requests high dose	Explain uncertainty and avoid unnecessary treatment burden.	High-dose supplementation as established adjunctive DMT.	Usually no additional MS-directed target.	Recent MRI-dominated signals do not establish reproducible clinical benefit.	[15,97,98,99,100,101,102,103,104,105,106]
Concern about cognitive decline without established impairment	Meaningful cognitive/social/intellectual engagement.	Promise to “build reserve” or prevent decline.	Engagement; cognitive testing only if clinically indicated.	Reasonable for participation/brain health; preventive cognitive efficacy unproven.	[107,108,109,110,111,113]
Established cognitive impairment	Assess and consider evidence-based cognitive rehabilitation.	Conflating rehabilitation with lifestyle enrichment.	Objective and functional cognitive goals.	Rehabilitation addresses a different therapeutic target.	[112]
Asks whether moderate alcohol is protective	No MS-directed prescription; follow general-health guidance.	Initiating/increasing alcohol for MS.	No MS therapeutic target.	Observational association does not justify intervention.	[17,18]
Several lifestyle targets coexist	Prioritize one or a few high-value feasible targets.	Universal comprehensive package by default.	Target-specific measures; sequence further changes.	Personalization is prioritization, not accumulation.	[9,10,11,12,13,16,54,55,56,57,114,115,116,117,118,119,120,121,122,123,124,125,126]
Intervention started but target does not change	Reassess barriers, support, dose and delivery.	Concluding target is ineffective before target engagement.	Direct target-engagement measure.	Failure of implementation differs from failure of a modified target.	[10,11]
Target changes but expected benefit does not occur	Reassess outcome, duration, magnitude and value of continuing.	Assuming target engagement guarantees benefit.	Verify maintenance and predefined outcome.	De-escalation or replacement may be appropriate.	[13]

Note. Evidence is grouped by its function in the decision: prognostic, intervention/target-modification, clinical-benefit, MS disease-course, guideline/implementation, safety/context, or author’s clinical synthesis. The clinical scenarios and proposed actions reflect the author’s evidence-informed interpretation and synthesis of the available literature and should not be understood as formally graded treatment recommendations or consensus guidance.

## Data Availability

No new data were created or analyzed in this study. Data sharing is not applicable to this article.

## Supplementary Materials

The following supporting information can be downloaded at: https://www.mdpi.com/article/10.3390/jpm16100523/s1, Supplementary Methods S1. Search Architecture and Evidence-Interpretation Framework.
