# Supplementary material for: From Association to Action: Personalizing Lifestyle Interventions in Multiple Sclerosis—A Critical Narrative Review
Source: J Pers Med. 2026 Oct 9;16(10):523. doi: 10.3390/jpm16100523 (PMC13706553; doi:10.3390/jpm16100523)
Supplement: Supplementary file 1 [file jpm-16-00523-s001.zip › jpm-4570042-supplementary.pdf]

Supplementary Methods S1
Search Architecture and Evidence-Interpretation Framework

From Association to Action: Personalizing Lifestyle Interventions in Multiple Sclerosis—A Critical Narrative Review

S1. Review type and purpose

This was a critical narrative review designed to evaluate post-diagnostic therapeutic actionability rather than to estimate pooled effects or provide an exhaustive systematic inventory. Searches and selection were structured and iterative but were not preregistered, and study screening did not follow a formal PRISMA process. This supplement reports the search architecture and the explicit rules used to select and interpret evidence; it should not be read as a retrospective claim of systematic-review methodology.

S2. Information sources and time window

The principal bibliographic source was PubMed/MEDLINE. Targeted bibliographic searches, reference-list examination of key reviews and pivotal studies, and verification against original publication records were used to identify additional relevant evidence. Searches were updated through 1 September 2026. No lower publication-date limit was imposed.

S3. Search architecture

The search architecture below documents the conceptual blocks used to structure iterative searching and evidence verification. Because the searches were not conducted under a prospectively fixed systematic-review protocol, these blocks should not be interpreted as fully reproducible Boolean search strings. No retrospective search syntax, filters or screening counts have been reconstructed.

Concept block	Core terms	Combined outcome terms	Evidence-type terms
Population	multiple sclerosis; clinically isolated syndrome where directly relevant	post-diagnostic; prognosis; disability; relapse; MRI; symptoms; cognition; quality of life	cohort; longitudinal; randomized; trial; systematic review; meta-analysis
Lifestyle domains	physical activity; exercise; smoking; obesity; weight; cardiometabolic; diet; sleep; stress; vitamin D; sunlight/UVR; cognitive reserve/engagement; alcohol	target-specific clinical and biological outcomes	observational association; exposure change; deliberate intervention
Translation	actionability; personalization; shared decision; behavior change	uptake; participation; adherence; fidelity; target engagement; maintenance; clinical benefit	implementation; feasibility; process evaluation; qualitative study
Complex interventions	multidomain; lifestyle program; digital intervention; self-management	component change; burden; sustainability; incremental benefit	randomized trial; pragmatic trial; process evaluation

S4. Evidence-selection principles

- Direct evidence in people with established MS was prioritized for post-diagnostic therapeutic claims.
- Susceptibility studies were retained only to distinguish etiological plausibility from post-diagnostic actionability.

- Large longitudinal cohorts, analyses of exposure change, randomized or deliberate intervention studies, systematic reviews and recent evidence capable of changing interpretation were prioritized.
- Mechanistic, MRI or biomarker studies were used to support biological plausibility, disease-activity signals or target modification, but not as substitutes for demonstrated clinical benefit.
- Implementation and qualitative studies were included when they informed uptake, adherence, fidelity, barriers, delivery, maintenance or feasibility.
- Evidence that did not directly resolve a decision could inform the author's clinical synthesis, provided that the synthesis was explicitly labelled and not presented as a validated recommendation.

S5. Evidence-interpretation dimensions

Each domain was interpreted using five dimensions: directness to the post-diagnostic question; study design and permissible causal inference; consistency across studies; verification and maintenance of change in the intended target; and the outcome actually measured. The therapeutic claim was then limited to the level supported by that outcome: general-health benefit, MS-relevant clinical benefit, or evidence relevant to MS disease-course modification.

The qualitative descriptors in Table 1 are intentionally not presented as formal GRADE ratings. "Replicated" or "consistent" indicates concordant evidence across more than one pertinent study or synthesis; "limited" indicates sparse, heterogeneous, indirect or short-duration evidence; "not established" indicates that available evidence is insufficient or inconsistent for the stated claim and does not prove absence of an effect.

S6. Operational distinctions used in the review

Term	Operational meaning
Uptake	The intervention is initiated or accepted.
Participation	Exposure to delivery, such as attendance, website use or program completion.
Adherence	The extent to which the participant follows the agreed intervention.
Fidelity	The extent to which the intervention is delivered as intended.
Target engagement	Verified change in the behavioral, environmental, biochemical or physiological exposure specified by the therapeutic hypothesis.
Clinical benefit	Improvement in the explicitly defined patient-relevant or disease-relevant outcome; assessed separately from target engagement.

S7. Methodological limitations

The review is vulnerable to selective availability of evidence and to author judgement in study prioritization and synthesis. It does not provide exhaustive screening counts, study-level risk-of-bias ratings or pooled effect estimates. These limitations are addressed by explicit separation of evidence types, conservative calibration of therapeutic claims, disaggregation of citations by evidentiary function and clear labelling of the author's clinical synthesis.
